# Removal of hexavalent chromium from aqueous solution by fabricating novel heteroaggregates of montmorillonite microparticles with nanoscale zero-valent iron

**DOI:** 10.1038/s41598-020-69244-z

**Published:** 2020-07-22

**Authors:** Yaru Yin, Chongyang Shen, Xiaoyuan Bi, Tiantian Li

**Affiliations:** Department of Soil and Water Sciences, China Agricultural University, Beijing, 100193 China

**Keywords:** Environmental sciences, Materials science, Nanoscience and technology

## Abstract

This study fabricated novel heteroaggregates of montmorillonite (Mt) microparticles with nanoscale zero-valent iron (nZVI) (Mt-nZVI) and examined the removal of Cr(VI) by the Mt-nZVI through batch experiments. Spherical nZVI particles were synthesized by the liquid phase reduction method, which were then attached on the flat Mt surfaces in monolayer. The fabricated Mt-nZVI had similar removal efficiency for Cr(VI) compared to the monodispersed nZVI particles, but was much greater than that of nZVI aggregates. The removal efficiency of Mt-nZVI increased with decreasing its dosage and increasing initial Cr(VI) concentration, whereas had insignificant change with solution pH. The removal of Cr(VI) by Mt-nZVI was well described by the pseudo second-order kinetics and the Langmuir equilibrium model. The removal was spontaneous and exothermic, which was mainly due to chemsorption rather than intra-particle diffusion according to calculation of change in free energy and enthalpy and Weber–Morris model simulations. X-ray diffraction and X-ray photoelectron spectroscopy analysis revealed that the adsorption was likely due to reduction of Cr(VI) to Cr(III) by Fe(0) and co-precipitation in the form of oxide-hydroxide of Fe(III) and Cr(III). The fabricated Mt-nZVI showed the promise for in-situ soil remediation due to both high removal efficiency and great mobility in porous media.

## Introduction

Nanoscale zero-valent iron (nZVI) has been shown to be very effective for treatment of various inorganic and organic pollutants in water in the past two decades^1,2^, due to its unique properties such as large specific surface area and high surface activity. However, the nZVI particles are readily to aggregate in water during the preparation and application because of attractive van der Waals force, high surface energy, and magnetic attractive force^3^. The aggregation reduces specific surface areas of the nZVI, which can significantly decrease its reactivity and efficiency for treatment of contaminants^4^. The aggregation also reduces the mobility of the nZVI in subsurface environments such as soil by sedimentation and straining at narrow pores^5^, and hence greatly limits its application for in-situ soil remediation.

Various techniques have been developed to prevent nZVI aggregation and enhance its dispersion^6,7^. For example, functional groups have been added on surfaces of nZVI via chemical modification (covalent or noncovalent functionalization)^8^. Adding functional groups increases surface charge, and consequently enhances electrostatic repulsion between nZVI particles in electrolyte solutions and their dispersion. In addition, polymers and surfactants have been used to stabilize the nZVI particles by inducing the steric repulsion between them^9^. However, it should be noted that adding functional groups or coating of these additives on nZVI particles masks the particle surfaces, which may decrease the efficiency of nZVI for contaminant treatment compared to bare monodispersed nZVI particles^10,11^.

The nZVI has also been frequently loaded on surfaces of templates such as polyacrylamide^12^, biochar^13^, Ca-alginate^14^, attapulgite^15^ and resins^16^ to increase its dispersion. Typically, the Fe(III) or Fe(II) was allowed to be homogeneously distributed on the template surfaces and then the nZVI particles were generated on the surfaces by adding NaBH_4_ to reduce the ferric ions to Fe(0)^17^. In addition to the aforementioned support materials, montmorillonite (Mt) is low-cost with a high cation exchange capacity and a large specific surface area^18,19^. Gu et al.^20^ used this method and prepared subnano-sized ZVI using smectite clay as templates. The Mt-nZVI has been used to adsorb and degrade a variety of contaminants such as heavy metals^4,21^ and organic contaminants^18,22^, which even showed higher performance in contaminant removal compared to bare nZVI due to synergetic effect between adsorption by Mt and removal by nZVI particles^17^.

It has to be noted that existing studies commonly prepared the nZVI-clay heteroaggregates by first treating clay particles with Fe cations^17^. Then the reduction was conducted to result in nZVI-clay by mixing Fe adsorbed clay particles with reducting agents. The sizes of the fabricated nZVI particles commonly were only several nanomaters. In this work, we developed a novel method to fabricate nZVI-clay. Specifically, we used liquid phase reduction method to first produce spherical nZVI particles. Then we allowed the nZVI particles to be attached on flat Mt surfaces in a monolayer via colloid-surface interactions. The sizes of the spherical nZVI particles can be tens of nanometers and the density of the nZVI particles on the Mt surfaces can be controlled by changing experimental conditions. Our fabricated Mt-nZVI can have great mobility in soil porous media because the attraction between the attached nZVI particles and collector surface could be eliminated by the strong repulsion between the Mt and surface^23,24^. Therefore, our fabricated Mt-nZVI will have great promise to be used for in-situ remediation of soil contaminants if it has great efficiency for contaminant removal in water. To the best of the authors’ knowledge, our study was the first to develop a method for fabricating the nano-micro structure by decorating spherical nanoparticles on flat microparticle surfaces in a monolayer.

The objective of this study was to fabricate the Mt-nZVI by attaching nZVI particles on Mt surfaces in a monolayer and examine its removal efficiency for Cr(VI) from aqueous solutions. Through conducting batch experiments, we showed that the Mt-nZVI had similar performance for removal of Cr(VI) in water compared to monodispersed nZVI particles, and the performance was much better than that of aggregated nZVI or Mt. The scanning electron microscope (SEM), X-ray diffraction (XRD), and X-ray photoelectron spectroscopy (XPS) examinations were conducted to reveal the mechanisms for the removal of Cr(VI) by the Mt-nZVI. The findings in this study showed the promise of use of Mt-nZVI for in-situ soil remediation.

## Results and discussion

### Characterization of Mt, nZVI, and Mt-nZVI

**Figure 1:**
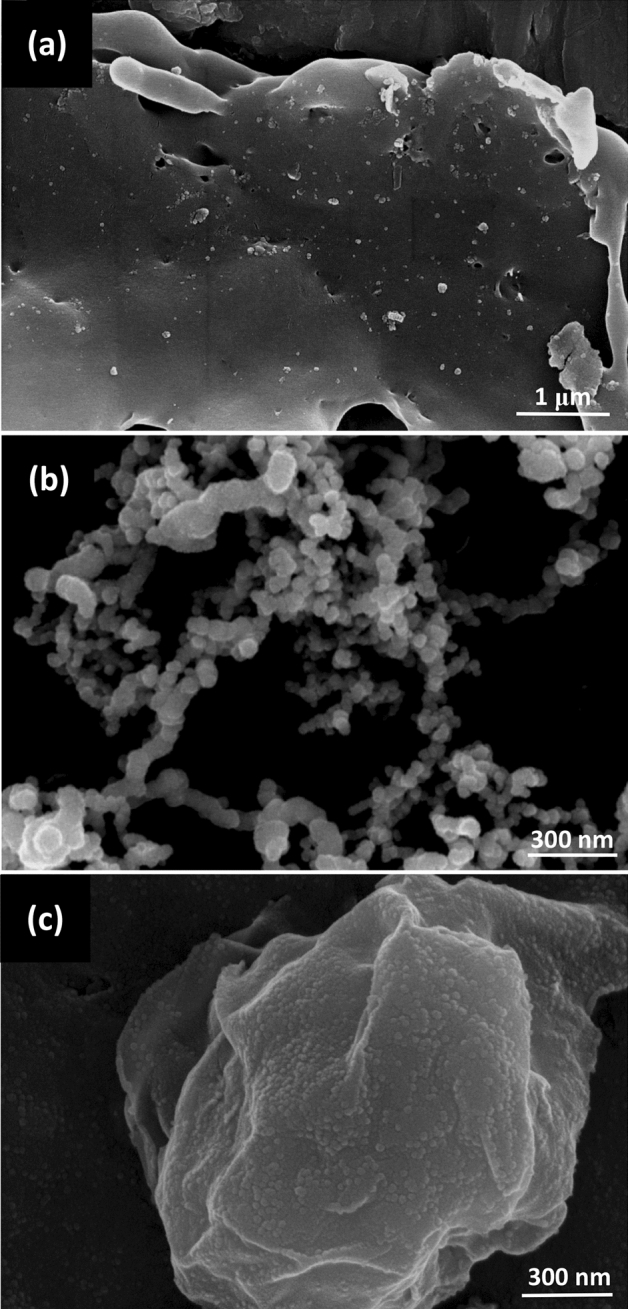
SEM images of (**a**) Mt, (**b**) nZVI, and (**c**) Mt-nZVI.

Figure 1 presents SEM images of Mt, nZVI particles, and Mt-nZVI. Mt was obtained from Zhejiang Sanding Technology Co. (Zhejiang, China) and its surface was relatively small and layered. The nZVI particles were roughly globular. Through sampling at least 100 nZVI particles from the SEM images, the average size of the nZVI particles was determined to be 50 nm. The nZVI particles were aggregated in deionized (DI) water as chain-like clusters due to van der Waals attraction and magnetic force^25^. By using Mt as a template, the aggregation was inhibited and the nZVI particles were evenly distributed on the Mt surfaces in monolayer. The attachment of nZVI particles on Mt surface in DI water was partly due to van der Waals attraction. As will be showing in the following by the XRD examinations, a very small fraction of nZVI was oxidized into Fe_2_O_3_. Attractive electrical double layer force existed between the positive charged iron oxide and negatively charged Mt surface, which can also assist the loading of the nZVI particles on the Mt surfaces.

**Figure 2:**
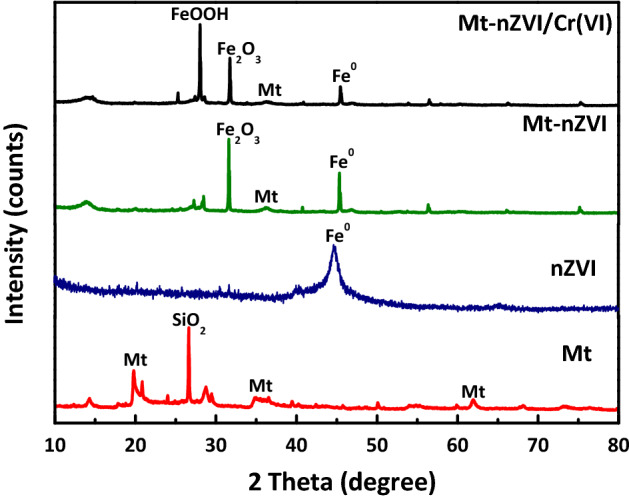
The X-ray diffractometer patterns of Mt, nZVI, Mt-nZVI, and Mt-nZVI after reaction with Cr(VI).

Figure 2 shows XRD patterns of the nZVI, Mt-nZVI, and Mt. The peak positions at 2θ = 44.67° for the XRD pattern of nZVI confirmed the existence of Fe^0^. There were no characteristic peaks of iron oxides observed for the XRD pattern of nZVI, implying that the nZVI particles were not oxidized and the purity of nZVI particles was high^1^. The XRD pattern of Mt-nZVI also showed an apparent peak of Fe^0^ and Mt, again verifying that the nZVI particles were loaded successfully onto the Mt. The small peak at 2θ of 32° indicated the presence of Fe_2_O_3_ due to oxidation of Fe^0^ when fabricating Mt-nZVI. Previous studies^26–28^ reported that iron nanoparticles have a core–shell structure, and the shell was due to rapid oxidation of the nascent nZVI to iron oxides while the core was Fe^0^. This structure can preserve the iron core against fast oxidation^18,28^. Therefore, the core–shell structure of the Mt-nZVI material has strong oxidation resistance. It has to be noted that the Mt-nZVI suspensions were very stable because the attraction between nZVI particles and nZVI particles or Mt surfaces were eliminated by the strong repulsions between the Mt surfaces.

### Adsorption of Cr(VI) onto Mt-nZVI

**Figure 3:**
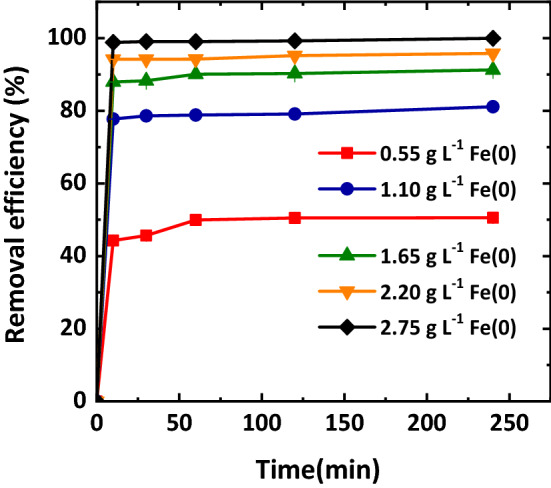
Removal efficiency of the Mt-nZVI with different concentrations of Fe loading. initial Cr(VI) concentration = 20 mg L^−1^; temperature = 25 °C, pH = 5.5, reaction time = 240 min. Error bars represent the standard deviations from triplicates.

Figure 3 presents the removal efficiency of Cr(VI) by Mt-nZVI with different loadings of nZVI particles. The concentration of Cr(VI) in the solution decreased very rapidly in the first 10 min and then slowly reduced. This indicates that most of the adsorption sites for Cr(VI) existed outside of the Mt-nZVI, which were easily accessible by the Cr(VI)^19^. The removal efficiency increased with increasing nZVI loading. Particularly, when the fraction of total nZVI mass per gram of Mt increased from 1 to 5 g g^−1^ (the corresponding concentrations of nZVI particles in the Mt-nZVI suspensions were 0.55 and 2.75 g L^−1^), the removal efficiency increased from 50.55 to 99.95%, respectively. This is expected because the nZVI is more effective to remove Cr(VI) compared to Mt and increasing the loading of nZVI increases active sites for Cr(VI) adsorption^16^.

**Figure 4:**
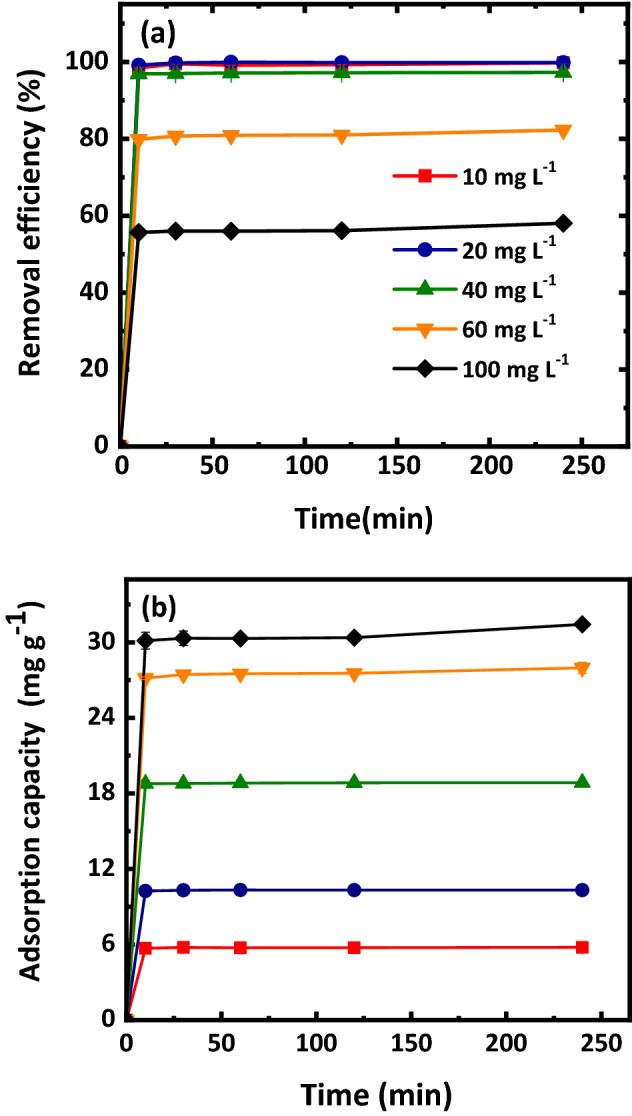
(**a**) Removal efficiencies and (**b**) adsorption capacity of Mt-nZVI at different initial concentrations of Cr(VI). pH = 5.5, temperature = 25 °C, reaction time = 240 min, concentration of Fe in Mt-nZVI suspension = 2.75 g L^−1^. Error bars represent the standard deviations from triplicates.

Figure 4 presents removal efficiency and adsorption capacity of Mt-nZVI at different initial Cr(VI) concentrations. The removal efficiency decreased with increasing Cr(VI) initial concentration. For example, when the initial concentration increased from 10 to 100 mg L^−1^, the removal efficiency decreased from 99.75 to 58.05%. This is because the adsorption sites on the Mt-nZVI were limited and the adsorption proceeded less completely with higher concentration of Cr(VI). In addition, increase in Cr(VI) concentration reduced the dissolution of Fe^0^^29^. This reduced the amount of Fe(II) formed due to reaction of Fe^0^ with H^+^ in water, and thus inhibited the reduction of Cr(VI) to Cr(III). Moreover, Zhang et al.^15^ illustrated that a large number of Cr(VI) anions were dispersed around Mt-nZVI and a Fe–Cr layer was formed instantaneously at high Cr(VI) concentration. This caused sealing of nZVI and prevention of electron transfer. Therefore, with increasing initial concentration of Cr(VI), the adsorption capacity increased whereas the removal efficiency increased.

Supplementary Fig. S1 compared the performance of Mt-nZVI with Mt, nZVI/H_2_O, and nZVI particles for removal of Cr(VI). The nZVI/H_2_O was most effective for removal of Cr(VI). Almost all Cr(VI) were removed from water using the nZVI/H_2_O. The nZVI/H_2_O had better performance than nZVI particles. This is because the nZVI particles were monodispersed in nZVI/H_2_O systems due to sonication before treatment of the Cr(VI). In contrast, aggregation of nZVI particles occurred when they were directly added into the Cr(VI) solution without sonication. Therefore, the nZVI/H_2_O had better performance than the nZVI particles. Note that hydration of nZVI particles may also increase the affinity of nZVI/H_2_O for adsorption of Cr(VI). The Mt had the worst performance. This is probably because the surface of Mt is mainly negatively charged due to isomorphous substitution, and electrostatic repulsion existed between Mt and Cr_2_O_7_^2−^^16^. Very limited positive charges existed at edge surfaces of Mt, which may cause the adsorption of Cr(VI)^30^. The removal efficiency was better for the nZVI/H_2_O than Mt-nZVI. This is because lower performance of Mt than monodispersed nZVI. However, the Mt-nZVI showed better performance than nZVI particles, indicating that the advantages for treatment of Cr(VI) due to monodispersion of nZVI particles on Mt surfaces compensated the limitation of using Mt which had a low removal efficiency^31^. The results in Supplementary Fig. S1 showed that if dosage of the Mt-nZVI was doubled, the removal efficiency reached > 99%, which was even greater than that of nZVI/H_2_O. Therefore, the Mt-nZVI showed great potential for in-situ remediation of contaminants in soil due to its high remove efficiency and great mobility in porous media with such unique nanomicro structures (data not shown).

Supplementary Fig. S2 presents the effect of solution pH on the removal efficiency of Cr(VI) by nZVI particles, nZVI/H_2_O, Mt, and Mt-nZVI after reaction of 4 h. The removal efficiency decreased significantly with increasing solution pH for the nZVI particles, in consistent with the observations in Shi et al.^4^ and Wu et al.^32^ This is likely because decrease of solution pH promoted the oxidation of iron and hence the reduction of Cr(VI) as well as the adsorption of Cr(VI). In addition, the nZVI particles are protonated to a higher extent under more acidic conditions, which are more favorable for adsorption of negatively charged Cr_2_O_7_^2−^^33^. With increasing solution pH, Cr_2_O_7_^2−^ competes with OH^−^ for adsorption sites on nZVI surfaces, which reduces Cr(VI) adsorption. Surprisingly, our results showed that the solution pH had little influence of the removal efficiency using the nZVI/H_2_O. The removal efficiency was very high (> 99%) at all solution pHs. This is probably because the ZVI particles were already oxidized during preparation of nZVI/H_2_O, causing excessive sites with positive charges for adsorption of Cr(VI) and little influence of solution pH. Due to a similar reason, the pH also had little influence on the remove efficiency by the Mt-nZVI.

It should be noted that it is important to examine the feasibility of the regeneration of the Mt-nZVI for its practical use. We have conducted additional experiments which showed excellent recyclability of Mt-nZVI. Specifically, the removal efficiency of Cr(VI) was 99.83% by adding 10 mL of Mt-nZVI suspension (0.55 g L^−1^ of Mt, 2.75 g L^−1^ of nZVI) into 20 mL of Cr(VI) solution (20 mg L^−1^) (i.e., the first circle of experiments). The removal efficiency only slightly changed to 97.65% at the second circle, indicating high recyclability of the Mt-nZVI.

### Kinetic and equilibrium adsorption of Cr(VI)

**Figure 5:**
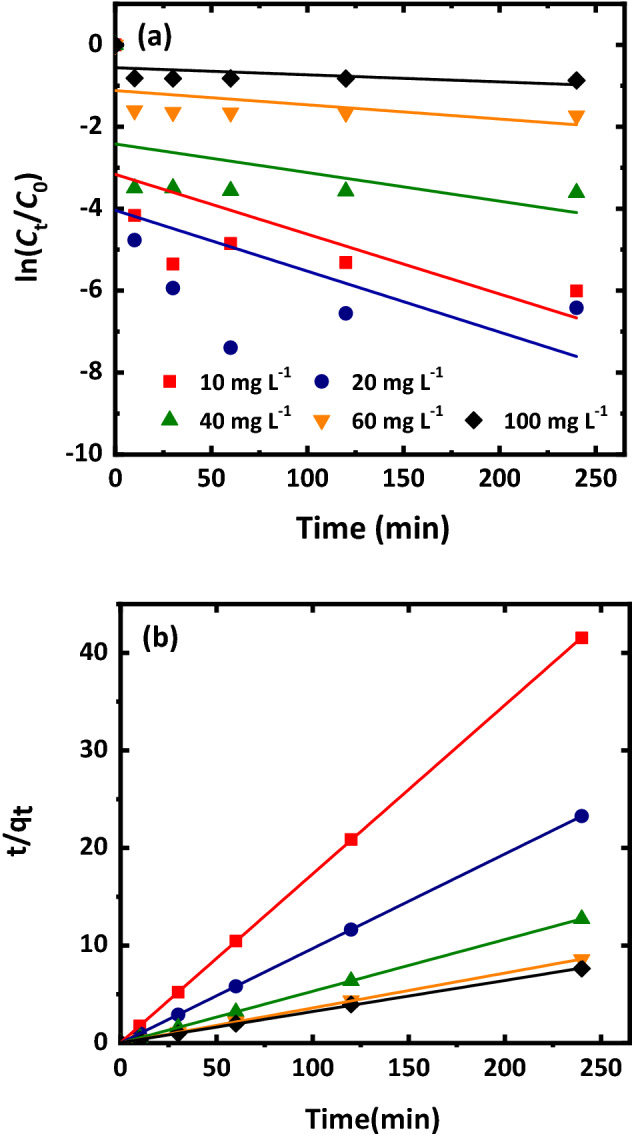
Plot of (**a**) pseudo-first-order and (**b**) pseudo-second-order kinetics for adsorption of Cr(VI) with different concentrations onto Mt-nZVI. *C*_0_ and *C*_t_ (mg L^−1^) are the concentration of Cr(VI) in the reaction solution before and at time *t* (min), respectively. pH = 5.5, reaction time = 240 min, concentration of Fe in Mt-nZVI suspension = 2.75 g L^−1^.

Figure 5 presents plots of using pseudo first- and second-order kinetic models for simulating adsorption of Cr(VI) onto Mt-nZVI at different initial concentrations. The simulated parameter values were shown in Supplementary Table S1. The pseudo second-order model simulated better than the pseudo first-order model. The removal of Cr(VI) was also found to follow pseudo-second-order kinetic interaction by using organo-montmorillonite supported nZVIs^32^, sodium dodecyl sulfate modified nZVIs^34^, and resin supported nZVIs^35^. The second-order kinetic interaction indicated that Cr(VI) adsorption was controlled by chemisorptions involving sharing or exchanging of electrons between the Cr(VI) and the Mt-nZVI^32,36^. The pseudo second-order rate constant (*k*_2_) increased with decreasing the initial concentration of Cr(VI). As mentioned previously, higher initial concentration of Cr(VI) caused the formation of Fe–Cr layer more rapidly. Such sealing of nZVI prevented subsequent adsorption of Cr(VI) and thus decreased the removal efficiency.

Supplementary Fig. S3 presents simulation of isotherm adsorption data using Langmuir and Freundlich model. The simulated equilibrium adsorption parameter values were presented in Table 1. The values of *R*^2^ for Langmuir and Freundlich models were 0.999 and 0.913, respectively, indicating that the Langmuir model provided a much better performance. The simulated maximum Cr (VI) adsorption capacity of Mt-nZVI obtained from the Langmuir isotherm model (31.646 mg g^−1^) was very similar to measured adsorption capacity *q*_m_ (31.445 mg g^−1^) when the initial concentration of Cr(VI) was 100 mg L^−1^. This indicated that the adsorption reached maximum at the highest initial concentration used in this study. The result also revealed that the Langmuir model did a very accurate prediction. The value of *q*_m_ decreased slightly with increase of temperature. Hence, adsorption of Cr(VI) onto Mt-nZVI was more favorable at lower temperature. The Langmuir adsorption indicates that the Cr(VI) was retained on Mt-nZVI surface in a monolayer, and there was no interaction or competition between Cr(VI) ions for adsorption on Mt-nZVI surfaces^37^. This adsorption also reflects that the retention of Cr(VI) by Mt-nZVI was likely due to chemisorption^38^. Supplementary Table S2 showed that the values of Δ*G* were negative at different temperatures. Accordingly, the adsorption of Cr(VI) by the Mt-nZVI was a favorable and spontaneous process^39^. This reaction process was endothermic since the calculated value of Δ*H* was positive. The value of Δ*S* was larger than zero, indicating an increase of entropy.

**Table 1:** Freundlich and Langmuir adsorption parameters at different temperatures.

Temperature (°C)	Langmuir			Freundlich		
	*q*_m_ (mg g^−1^)	*K*_L_	*R*^2^	*k*_F_	*n*	*R*^2^
25	31.646	105.333	0.999	36.413	4.885	0.913
35	22.723	36.667	0.995	23.571	4.854	0.977

*q*_m_ denotes the maximum adsorption capacity.

*K*_L_ denotes the Langmuir isotherm constants.

*K*_F_ denotes the Freundlich isotherm constants.

*n* denotes the adsorption intensity.

*R*^2^ denotes the linear regression coefficient.

**Figure 6:**
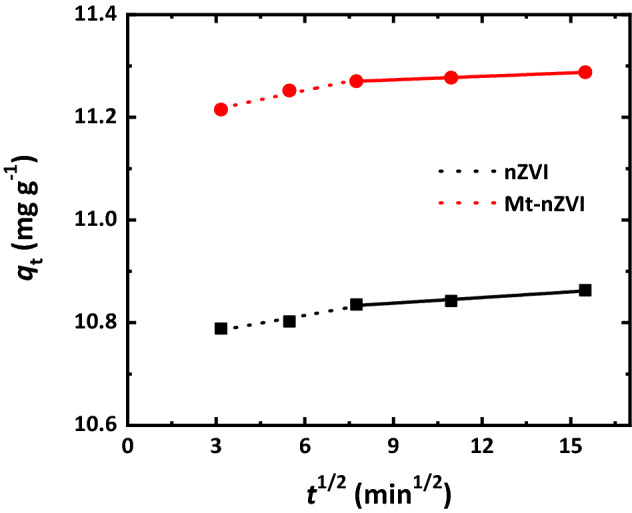
Simulation of remval of Cr(VI) by nZVI and Mt-nZVI using intra-particle diffusion model. The Fe concentrations of nZVI and Mt-nZVI suspensions were 1.1 g L^−1^. The Mt concentrations of Mt-nZVI was 0.22 g L^−1^. pH = 5.5, initial Cr(VI) concentration = 20 mg L^−1^, temperature = 25 °C, reaction time = 240 min.

The Weber and Morris intra-particle diffusion model was also used to interpret the Cr(VI) adsorption mechanism. This model is written as^40^

1 $$ q_{{\text{t}}} = k_{{\text{p}}} t^{0.5} + C $$

where *q*_t_ (mg g^−1^) is the amount of Cr(VI) adsorbed at time *t* (min), *k*_p_ (mg g^−1^ min^−0.5^) is intraparticle diffusion rate constant and *C* (mg g^−1^) is intercept at the ordinate related to the boundary layer thickness. If the plot of *q*_t_ versus *t*^0.5^ is a straight line and the line passes through the origin, the adsorption process is only controlled by intra-particle diffusion. However, if the line does not pass the origin or multi-linear features exist between the *q*_t_ and *t*^0.5^, the adsorption process is regarded to be controlled by two or more steps during the adsorption process^41^. The results in Fig. 6 indicated existence of two simulated lines for the plot of *q*_t_ versus *t*^0.5^, and none of them passed through the origin. Therefore, the removal process could be described by multi-diffusion steps^37^. When the adsorbent was added to the Cr(VI) solution, the initial rate of removal was rapid (e.g., film diffusion), and then the removal gradually slowed down (e.g., intra-particle diffusion)^42^. The diffusion rate constant in the first stage was higher for the Mt-nZVI than nZVI (Supplementary Table S3), which might be attributed to the existence of more active sites on surfaces of Mt-nZVI, making the reaction more rapid and efficient^43^.

Table 2 compared the removal efficiencies for Cr(VI) using nZVI particles prepared with different methods. Toli et al.^44^ used green tea (GT) extract instead of NaBH_4_ to reduce Fe(III), and macroreticular resin (R) as porous support to prepare the nZVI composite materials (R-nFe). The removal efficiency of the R-nFe for treating the Cr(VI) was only 29.8% in 24 h. Similarly, Soliemanzadeh et al.^37^ used the green tea extracts to prepare bentonite (Bt)-loaded nZVI (Bt-nZVI). They showed that the maximum adsorption capacity of Bt-nZVI for Cr(VI) was as high as 60.56 mg g^−1^, which was higher than that of Mt-nZVI (31.646 mg g^−1^) in our study. However, the maximum adsorption capacity of Mt-nZVI was much higher than those of Bt-nZVI prepared by Shi et al.^4^ (16.67 mg g^−1^) and Zhang et al.^45^ (16 mg g^−1^) using the liquid phase reduction method as used in our study. In addition, the removal efficiency of Mt-nZVI was as high as 99.75%, which is much higher than those of the composite material of Mt and nZVI by Wang et al.^46^ and Zhang et al.^1^.

**Table 2:** Removal efficiencies for Cr(VI) using different nZVI composite materials.

Materials	Preparation method	*q*_e_ (mg g^−1^)	Removal efficiency	References
Mt-nZVI	Mt + nZVI(FeCl_2_·4H_2_O + NaBH_4_)	31.65	99.75% in 240 min	–
Bt + nZVI	Liquid phase reduction method	16.67	99%	^4^
Bt + nZVI	Liquid phase reduction method	–	50% in 180 min	^47^
Bt + nZVI	Liquid phase reduction method	16	92.55% in 20 s	^46^
Starch-stabilized Mt + nZVI	Liquid phase reduction method	6.85	5.56% in 5 min	^1^
R-nFe	Resin + Fe(III) solution + green tea extracts	–	29.8% in 24 h	^45^
Bt-nZVI	Bt + FeSO_4_·7H_2_O + green tea extracts	66	–	^39^

*q*_e_ denotes the amount of Cr(VI) adsorbed at equilibrium.

R-nFe denotes the nZVI composite material (R-nFe) with macroporous resin (R) as porous support.

Bt-nZVI denotes the bentonite (Bt)-loaded nZVI (Bt-nZVI).

### Adsorption mechanisms

**Figure 7:**
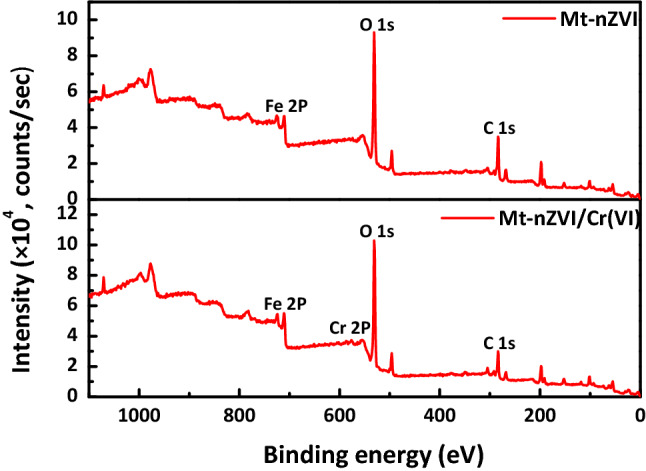
XPS spectra of Mt-nZVI before reaction and after reaction with Cr(VI).

Figure 7 presents the XPS spectra of Mt-nZVI before and after reaction with Cr(VI). Two peaks at binding energies of 711.3 eV and 724.9 eV existed, which could be attributed to the 2p3/2 and 2p1/2 peaks of Fe(III), respectively^21^. Therefore, a fraction of Fe(0) has been oxidized to Fe(III) species in fresh Mt-nZVI, in agreement with the observations by XRD analysis in Fig. 2. Figure 7 shows that, after reaction with Cr(VI), new peaks emerged at 577 and 586 eV for the spectra of Mt-nZVI, indicating the existence of Cr(III) oxide and chromium hydroxide^32^. Consequently, the chromium that was adsorbed onto Mt-nZVI was reduced to Cr(III). The reduction of Cr(VI) may occur after the adsorption of Cr(VI) onto Mt-nZVI, but may also occur in the solution. Specifically, oxidation of nZVI resulted in dissolved Fe(II) in the solution^47^ and the further transformation of Fe(II) to Fe(III) could cause reduction of Cr(VI). Notably, the XRD patterns in Fig. 2 showed that the characteristic peak of FeOOH (2θ = 26.98°) emerged for the spectra of Mt-nZVI after reaction with Cr(VI). These results indicate that the reaction products may be retained on the Mt-nZVI surfaces via co-precipitation in the form of oxide-hydroxide of Fe(III) and Cr(III), as revealed by a number of previous studies^4,16^. It is worthwhile mentioning that Mt is a good conductor^48,49^. The Mt may assist the transfer of electrons from the nZVI to Cr(VI) and accordingly the reduction of Cr(VI) to Cr(III).

## Conclusions

We have developed a novel method for fabrication of heteroaggregates of Mt with nZVI particles. The nZVI particles were first synthesized via liquid phase reduction method and then attached on Mt surfaces in a monolayer. The sizes of the nZVIs particles and the concentration of the attached nZVI particles on the Mt could be controlled by varying experimental conditions. We showed that the Mt-nZVI had similar efficiency for removal of Cr(VI) from water compared to monodispersed nZVI particles, and the removal efficiency was significant greater than aggregated nZVI particles. The XRD and XPS examinations showed that Cr(III) and FeOOH emerged on Mt-nZVI surfaces after reaction with Cr(VI), indicating that co-precipitation of chromium and Fe [in the form of oxide-hydroxide of Fe(III) and Cr(III)] likely was the main removal mechanism. The removal process followed the pseudo second-order kinetic interaction and the isotherms were well described by Langmuir model. The Weber-Morris diffusion model simulations demonstrated that the removal kinetic was influenced both by film diffusion and intra-particle diffusion. The thermodynamic calculations indicated that the removal was due to chemisorption, which was spontaneous and endothermic. Because the Mt-nZVI has high mobility in soil porous media due to its unique nano-micro structure, the Mt-nZVI shows the promise of in-situ treatment of contaminants in soil.

## Materials and methods

### Synthesis of nZVI and Mt-nZVI

nZVI was synthesized using the well-known liquid phase reduction method^50^. Briefly, 21.36 g of FeCl_2_·4H_2_O were added into ethanol-water solution containing 96 mL anhydrous ethanol and 24 mL DI water to obtain ferrous solution. 12.2 g of NaBH_4_ powder were dissolved in 400 mL of DI water, and the resulting NaBH_4_ solution was dropped into the aforementioned ferrous solution with stirring. With addition of NaBH_4_, the ferrous solution immediately became black due to reduction of Fe(II) to Fe(0). The suspension was shaken for 2 h at a speed of 180 r min^−1^. The nZVI particles were separated by vacuum filtration of the suspensions using 0.22 μm filter. The collected nZVI particles were washed using 99% ethanol and then dried at 85 °C for 10 h.

Mt powder was purchased from Zhejiang Sanding Technology Co. (Zhejiang, China). The suspensions of Mt particles with diameters of 1–2 μm were prepared through sedimentation^51^. The nZVI particle suspension was prepared by dissolving the nZVI particle in DI water. Both nZVI and Mt suspensions were sonicated at least 15 min for ensure the monodispersity, and the Mt suspension was carefully transferred into the nZVI suspension by stirring. The suspensions with Mt and nZVI particles were shaken for 12 h at a speed of 200 r min^−1^ to obtain the Mt-nZVI. By changing the ratio of concentration of nZVI and Mt suspensions, we obtained Mt-nZVI with different concentrations of ZVI particles attached on the Mt surface. The morphology and structure of the prepared nZVI and Mt-nZVI were characterized by SEM (Hitachi S-4800, Japan). The identification of crystalline phases of the nZVI and Mt-nZVI was conducted by XRD analysis on a Bruker X-ray powder diffractometer (model D8-Discover). Specifically, the samples were prepared for XRD analysis by first drying and then grinding into powder, and the operations were implemented using Cu-Ka radiation source at 40 kV and 40 mA. XPS was applied to analyze valence variations of Fe and Cr after reaction of the adsorbent with Cr(VI). XPS was measured via a PHI qUANTERA II X-ray photoelectron spectrometer with using monochromatized Al Kα radiation (1,486.92 eV). Sprinkle powder on the surface of the sticky strip was adopted for analysis. The binding energy of C 1 s was shifted to 284.8 eV as an internal reference.

### Batch experiments

Through conducting batch experiments, the effects of different factors on the removal efficiency of Cr(VI) by Mt-nZVI were examined including concentration of Fe loading on Mt surfaces, solution pH, and initial concentration of Cr(VI). The performance of Mt-nZVI with Mt and bare nZVI were compared. The equilibrium and kinetic sorption were also investigated.

Cr(VI) solution was prepared by dissolving K_2_Cr_2_O_7_ in DI water. To examine the influence of concentration of Fe loading on Mt surfaces, different amounts of nZVI particles (0, 0.11, 0.22, 0.33, 0.44, and 0.55 g) were added into 100 mL DI water, and then 100 mL of Mt solution (1.1 g L^−1^) was added into the nZVI suspensions to obtain the Mt-nZVI suspensions using the aforementioned method. The concentration of Mt for the Mt-nZVI suspensions was 0.55 mg L^−1^. The concentrations of nZVI particles for the Mt-nZVI suspensions were 0, 0.55, 1.1, 1.65, 2.2, 2.75 g L^−1^, corresponding to of 0, 1, 2, 3, 4, 5 g g^−1^ of the fraction of nZVI mass per gram of Mt, respectively. 10 mL of the prepared Mt-nZVI suspension with different concentrations of nZVI was added into 20 mL of Cr(VI) solution (20 mg L^−1^). The mixture was shaken at 25℃ at 180 rpm. The supernatant was collected at 10, 30, 60, 120, 240 min and filtrated through a 0.45 μm membrane to examine the adsorption kinetics. The concentration of Cr(VI) in the supernatant was determined using an UV–Vis spectrophotometry (DU 800, Beckman Intruments, Inc., Fullerton, California) via the diphenylcarbazide spectrophotometric method^52^. The removal efficiency of Cr(VI) (*η*, %) and adsorption capacity (*q*_t_, mg g^−1^) were calculated using the following expressions

2 $$ \eta = \frac{{C_{0} - C_{t} }}{{C_{0} }} \times 100{ } $$

3 $$ q_{t} = \frac{{\left( {C_{0} - C_{t} } \right)V}}{m} $$

where *C*_0_ (mg L^−1^) is the initial Cr(VI) concentration; *C*_t_ (mg L^−1^) is the concentration of Cr(VI) at time *t* (min); *q*_t_ (mg g^−1^) is the absorption capacity; *V* (mL) is volume of solution; *m* (g) is mass of the adsorbent.

As will be shown later in the paper, the removal efficiency increased with increasing the concentration of nZVI loading. The removal efficiency reached maximum for the Mt-nZVI with a 5 g g^−1^ of the fraction of total nZVI mass per gram of Mt, which were thus used for the following batch experiments. The procedures of these batch experiments followed the same produce as shown above expect that some experimental conditions were varied. Specifically, 10 mL of Mt-nZVI suspension (0.55 g L^−1^ of Mt) was added into 20 mL of Cr(VI) solution (0, 10, 20, 40, 60 and 100 mg L^−1^) at pH 5.5 to investigate the effect of initial Cr(VI) concentration. To compare the performance of Mt, nZVI, and Mt-nZVI, 20 mL of Cr(VI) solution (20 mg L^−1^) at pH 5.5 was mixed with 5 ml of Mt-nZVI suspension (0.55 g L^−1^ of Mt, 2.75 g L^−1^ of nZVI), 0.011 g nZVI particles, 5 ml of nZVI suspension (2.75 g L^−1^), or 5 ml of Mt suspension (0.55 g L^−1^). The concentrations of Mt and nZVI for all resulting mixtures were kept at 0.11 g L^−1^ and 0.55 g L^−1^, respectively. We used both nZVI particles and nZVI suspension to understand the effect of hydration on the removal of Cr(VI) by the nZVI. The nZVI suspension was denoted as nZVI/H_2_O later in the paper. Note that we have also compared the performance of Mt, nZVI, and Mt-nZVI at Mt and nZVI concentrations of 0.22 and 1.1 g L^−1^, respectively. To investigate the influence of solution pH on the removal efficiency, 20 mL of Cr(VI) solution (20 mg L^−1^) at different pHs (3, 5, 5.5, 7, 9, 11) was mixed with 5 mL Mt-nZVI suspensions (0.55 g L^−1^ of Mt, 2.75 g L^−1^ of nZVI), 0.011 g nZVI particles, 5 ml of nZVI suspension (2.75 g L^−1^), or 5 ml of Mt suspension (0.55 g L^−1^).

To examine the adsorption kinetics of the Cr(VI) by the Mt-nZVI, 10 mL of Mt-nZVI suspension (0.55 g L^−1^ of Mt, 2.75 g L^−1^ of nZVI) was added to 20 mL of Cr(VI) solution at different concentrations (0, 10, 20, 40, 60, 100 mg L^−1^). The supernatant was collected at time 0, 10, 30, 60, 120, and 240 min for determining the concentration of Cr(VI). The equilibrium experiments followed the same procedure of the kinetic experiments to obtain isotherm data. However, we only examined the concentrations of Cr(VI) in the supernatant at 240 min. As will be shown later in the paper, the reaction reaches equilibrium after 240 min. The pseudo-first-order kinetic and pseudo-second-order kinetic models were used to simulate the data, which were written as

4 $$ {\ln}\frac{{C_{{\text{t}}} }}{{C_{0} }} = - k_{1} t $$

5 $$ \frac{t}{{q_{{\text{t}}} }} = \frac{1}{{k_{2} q_{{\text{e}}}^{2} }} + \frac{t}{{q_{{\text{e}}} }} $$

where *k*_1_ (min^−1^) and *k*_2_ (g mg^−1^ min^−1^) represent the pseudo-first-order rate constant and pseudo-second-order rate constant, respectively, *q*_e_ and *q*_t_ (mg g^−1^) are the amount of Cr(VI) adsorbed at equilibrium and at time *t* (min), respectively. The Langmuir and Freundlich isotherm models were used to simulate the isotherms, which were given by

6 $$ \frac{{C_{{\text{e}}} }}{{q_{{\text{e}}} }} = \frac{1}{{q_{{\text{m}}} K_{{\text{L}}} }} + \frac{{C_{{\text{e}}} }}{{q_{{\text{m}}} }} $$

7 $$ {\ln}q_{{\text{e}}} = {\ln}K_{{\text{F}}} + \frac{1}{n}{\ln}C_{{\text{e}}} $$

where *C*_e_ (mg L^−1^) is the equilibrium concentration, *q*_e_ (mg g^−1^) and *q*_m_ (mg g^−1^) are the equilibrium absorption capacity and the maximum adsorption capacity, respectively, *K*_L_ (g mg^−1^) is a constant related to adsorption capacity, *K*_F_ is the Freundlich isotherm constants related to adsorption, *n* is the adsorption intensity.

To further reveal the mechanisms of adsorption of Cr(VI) by Mt-nZVI, the values of thermodynamic parameters including change in free energy Δ*G* (kJ mol^−1^), enthalpy change Δ*H* (kJ mol^−1^), and change in entropy Δ*S* (J mol^−1^ K^−1^) were determined using the following expressions

8 $$ {\Delta }G = - RT{\ln}K_{1} $$

9 $$ {\Delta }H = R\frac{{T_{2} T_{1} }}{{T_{2} - T_{1} }}{\ln}\frac{{K_{2} }}{{K_{1} }} $$

10 $$ {\Delta }S = \frac{{{\Delta }H - {\Delta }G}}{{T_{1} }} $$

where *R* is molar gas constant, *T*_i_ is absolute temperature, *K*_i_ is equilibrium adsorption constant. To obtain the values of Δ*H*, we conducted the equilibrium adsorption experiments at both 25 °C and 35 °C.

## Supplementary information

Supplementary information.

## Data Availability

All data generated or analyzed during this study are included in this article (and its Supplementary Information File).
