# Supplementary material for: Removal of hexavalent chromium from aqueous solution by fabricating novel heteroaggregates of montmorillonite microparticles with nanoscale zero-valent iron
Source: Sci Rep. 2020 Jul 22;10:12137. doi: 10.1038/s41598-020-69244-z (PMC7376241; doi:10.1038/s41598-020-69244-z)
Supplement: Supplementary file 1 — Supplementary information. [file 41598_2020_69244_MOESM1_ESM.docx]

**Supplementary Material**

Removal of hexavalent chromium from aqueous solution by fabricating novel heteroaggregates of montmorillonite microparticles with nanoscale zero-valent iron

Yaru Yin,^†^ Chongyang Shen,^†,*^ Xiaoyuan Bi,^†^ Tiantian Li^†^

^†^Department of Soil and Water Sciences, China Agricultural University, Beijing 100193, China.

*Corresponding author Chongyang Shen: Phone, +86 10 62733596; Fax, +86 10 62733596; Email, [chongyang.shen@cau.edu.cn](mailto:chongyang.shen@cau.edu.cn).

**Supplementary** **Table S1.** Kinetics constants for the removal of Cr(VI) at different initial Cr (VI) concentration.

| Initial Cr (VI) concentration  (mg L-1) | Pseudo first-order model | | Pseudo second-order model | | |
| --- | --- | --- | --- | --- | --- |
|  | *k*1 (min-1) | *R*2 | *k*_2_ (g mg-1 min-1) | *q*e (mg g-1) | *R*2 |
| 10 | 0.015 | 0.369 | 0.674 | 5.780 | 1.000 |
| 20 | 0.015 | 0.254 | 0.538 | 10.320 | 1.000 |
| 40 | 0.001 | 0.192 | 0.362 | 18.868 | 1.000 |
| 60 | 0.004 | 0.217 | 0.027 | 28.011 | 1.000 |
| 100 | 0.002 | 0.214 | 0.012 | 31.445 | 1.000 |

*k*_1_ denotes the pseudo first-order rate constant.

*k*_2_ denotes represents the pseudo second-order rate constant.

*q*_e_ denotes the amount of Cr(VI) adsorbed at equilibrium.

*R^2^* denotes the linear regression coefficient.

**Supplementary Table S2.** Thermodynamic parameters at different temperatures.

| Temperature (℃) | *K*_i_ | Δ*G* (kJ mol^-1^) | Δ*S* (J mol^-1^ K^-1^) | Δ*H* (kJ mol^-1^) |
| --- | --- | --- | --- | --- |
| 25 | 105.333 | -15410.302 | 60.164 | 2518.685 |
| 35 | 36.667 | -12217.970 | 47.846 | 2518.685 |

*K*_i_ denotes the equilibrium adsorption constant.

Δ*G* denotes the change in free energy.

Δ*S* denotes the change in entropy.

Δ*H* denotes the enthalpy change.

**Supplementary Table S3.** The Intra-particle diffusion model parameters for nZVI and Mt-nZVI suspensions.

| Intra-particle diffusion | Parameters | nZVI | Mt-nZVI |
| --- | --- | --- | --- |
| first stage | k_p_ (mg g^-1^ min^-0.5^) | 0.010 | 0.0120 |
|  | C (mg g^-1^) | 10.75 | 11.180 |
|  | R^2^ | 0.945 | 0.945 |
| second stage | k_p_ (mg g^-1^ min^-0.5^) | 0.004 | 0.002 |
|  | C (mg g^-1^) | 10.805 | 11.253 |
|  | R^2^ | 0.965 | 0.999 |

*k*_p_ denotes the intraparticle diffusion rate constant.

*C* denotes the intercept at the ordinate.

**Supplementary Figure S1.** Removal efficiency of Mt, nZVI/H_2_O, nZVI, and Mt-nZVI. The Fe concentrations of nZVI/H_2_O, nZVI, and Mt-nZVI suspensions were 0.55 g L^-1^ and 1.1 g L^-1^ in (a) and (b), respectively. The Mt concentrations of Mt and Mt-nZVI concentrations were 0.11 and 0.22 g L^-1^ in (a) and (b), respectively. pH=5.5, initial Cr(VI) concentration=20 mg L^-1^, temperature=25℃, reaction time=240 min. Error bars represent the standard deviations from triplicates.

**Supplementary Figure S2.** Effect of pH on the removal efficiency of Mt, nZVI/H_2_O, nZVI, and Mt-nZVI. pH=5.5, initial Cr(VI) concentration=20 mg L^-1^, temperature=25℃, pH=5.5, reaction time=240 min, concentration of Fe in Mt-nZVI suspension=2.75 g L^-1^. Error bars represent the standard deviations from triplicates.

**
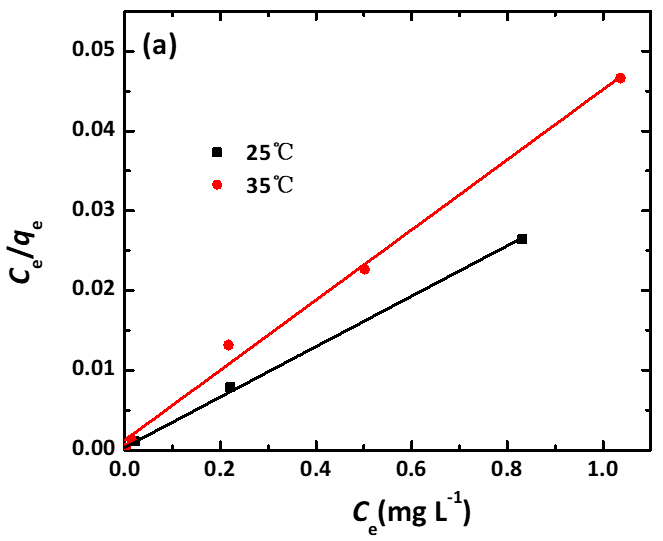

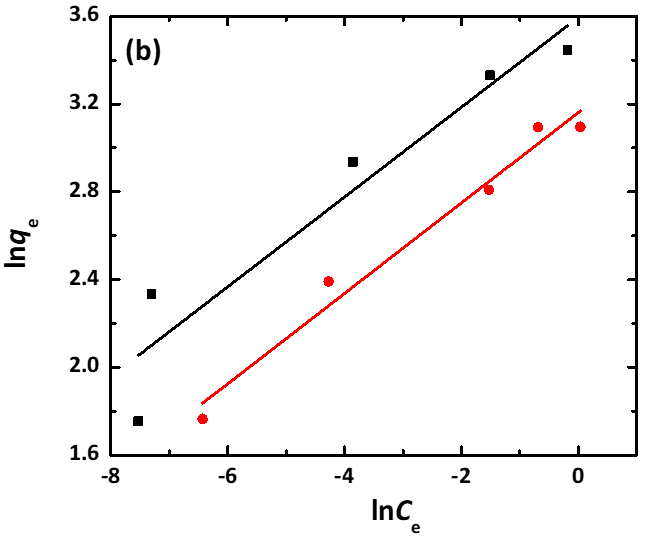
**

**Supplementary Figure S3.** (a) Langmuir and (b) Freundlich isotherms for Cr(VI) adsorption on Mt-nZVI at different temperatures. pH=5.5, equilibrium time=240 min, concentration of Fe in Mt-nZVI suspension=2.75 g L^-1^.
